# Obsessive–compulsive symptoms and information seeking during the Covid-19 pandemic

**DOI:** 10.1038/s41398-021-01410-x

**Published:** 2021-05-21

**Authors:** Alisa M. Loosen, Vasilisa Skvortsova, Tobias U. Hauser

**Affiliations:** 1 Max Planck UCL Centre for Computational Psychiatry and Ageing Research, London, UK; 2 Wellcome Centre for Human Neuroimaging, University College London, London, UK

**Keywords:** Psychiatric disorders, Depression

## Abstract

Increased mental-health symptoms as a reaction to stressful life events, such as the Covid-19 pandemic, are common. Critically, successful adaptation helps to reduce such symptoms to baseline, preventing long-term psychiatric disorders. It is thus important to understand whether and which psychiatric symptoms show transient elevations, and which persist long-term and become chronically heightened. At particular risk for the latter trajectory are symptom dimensions directly affected by the pandemic, such as obsessive–compulsive (OC) symptoms. In this longitudinal large-scale study (*N* = 406), we assessed how OC, anxiety and depression symptoms changed throughout the first pandemic wave in a sample of the general UK public. We further examined how these symptoms affected pandemic-related information seeking and adherence to governmental guidelines. We show that scores in all psychiatric domains were initially elevated, but showed distinct longitudinal change patterns. Depression scores decreased, and anxiety plateaued during the first pandemic wave, while OC symptoms further increased, even after the ease of Covid-19 restrictions. These OC symptoms were directly linked to Covid-related information seeking, which gave rise to higher adherence to government guidelines. This increase of OC symptoms in this non-clinical sample shows that the domain is disproportionately affected by the pandemic. We discuss the long-term impact of the Covid-19 pandemic on public mental health, which calls for continued close observation of symptom development.

## Introduction

The global coronavirus SARS-CoV-2 (Covid-19) pandemic has created a situation of severe uncertainty and isolation, fuelled by disruptions in finance, politics, social life and healthcare. As such, it constitutes an immense psychological challenge, especially for people’s mental health.

First studies have reported adverse psychological consequences of the pandemic among people with and without pre-existing mental-health conditions. Patients with anxiety, depression, bipolar disorders, schizophrenia^1–3^ and obsessive–compulsive disorder (OCD)^4^ were reported to experience a pandemic-related increase in symptoms. Likewise, the general public reported worsened mental health with a rise primarily seen in anxiety and depression levels^5–10^.

Obsessive–compulsive (OC) symptoms are likely to be disproportionately affected by the pandemic^11^ as many of them revolve around contamination, infectious illnesses, and causing harm^12^. First evidence suggested a worsening of symptoms in OCD patients during the pandemic^4,13–16^, although the results are somewhat inconsistent (cf.^14,17,18^), which may be driven by the heterogeneity of the disorder or differences in underlying comorbidities. How OC symptoms developed in non-patient populations during the pandemic is, however, to the best of our knowledge, unknown. Research conducted on previous health crises, such as the HIV/AIDS epidemic in the 1980s has shown that health campaigns could influence the occurrence of epidemic-related OCD-symptomatic behaviour in individuals with no reported history of psychiatric disorders^19–21^. These findings raise the concern that Covid-19 campaigns may have a similar detrimental effect on the general public.

In addition, elevated levels of psychiatric symptoms following a stressful time, such as the pandemic^8,22,23^, are common. Decades of research demonstrated that mental health worsens after stressful events^24,25^. However, importantly, psychiatric levels usually improve after a reasonable time without inflicting long-term impairments^26–28^. This adaptation process has been attributed to coping and (re-)appraisal strategies^29^. A failure of this process, however, may lead to long-term adverse consequences and chronic mental-health problems^26,27^. It is thus critical to not only assess momentary increases in psychiatric symptoms but to also investigate the long-term trajectories of these symptoms.

Here, we conducted a large-scale, non-clinical, longitudinal study investigating OC, anxiety and depression symptoms measured as self-reported dimension scores during the Covid-19 pandemic. We tracked these psychiatric scores over a period of several months and show that OC symptoms in this general UK public sample increase, whilst anxiety levels plateau and depression levels diminish. Moreover, we investigated how these symptoms impacted participants’ Covid-related information seeking and adherence to governmental guidelines, based on the hypothesis that OC symptoms are associated with excessive information seeking^30–34^. We show that rising OC symptoms in the general public are linked to pandemic-related information seeking, which in turn promotes higher guideline adherence.

## Materials and methods

### Study design and procedure

**Fig. 1:**
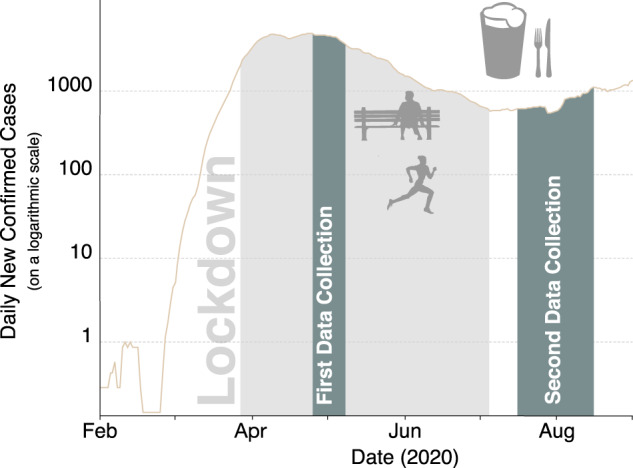
Longitudinal data collection of mental health symptoms. The first data collection took place from the 24th of April to the 7th of May. Shown is the log-average of daily new confirmed Covid-19 cases, using the rolling 7-day average in the UK from the 1st of February 2020 to the 1st of September 2020. Lockdown was formally enforced on the 26th of March. The ease of lockdown commenced on the 10th of May when people were encouraged to visit parks again and engage in unlimited outdoor exercise. On the 4th of July pubs and restaurants were among the last businesses to come out of lockdown. The second data collection took place from the 15th of July until the 15th of August, after the lockdown restrictions had been lifted. The pandemic curve has been plotted on the basis of data maintained by Our World in Data^63^.

We conducted a longitudinal online study of the general public, collecting data at two time points throughout the first pandemic wave (April to August 2020; Fig. 1). At both time points, participants reported OC^35^, anxiety, and depression symptoms^36^ using standardised questionnaires. In addition, we recorded participants’ Covid-19-related information seeking with a new scale (cf. below) as well as whether they had received a positive Covid-19 test or/and had essential worker status (as defined by the UK government) during or before data collection. At the first time point (T1), participants also reported their baseline news and social media consumption and completed a cognitive ability assessment^37^ serving as an IQ estimation. At the second time point (T2), we additionally measured people’s adherence to Covid-19 guidelines as instructed by the UK government.

Data collection for T1 lasted from the 24th of April 2020 (first pilot) until the 7th of May 2020. This was approximately at the peak of the first pandemic wave in the UK (Fig. 1). The second data collection (T2) directly followed the largest lift in pandemic-related restrictions in the UK (i.e. reopening of restaurants and pubs), which allowed us to assess the adaptation of mental-health symptoms to a substantial environmental change. The T2 data were collected between the 15th of July 2020 and the 15th of August 2020.

### Participants

We recruited participants living in the UK via the Prolific recruiting service (https://www.prolific.co/). All subjects were over 18 and gave their informed consent before starting the study. There were no other recruitment restrictions to gain a representative sample of the general public. The study was approved by University College London’s Research Ethics Committee.

A total of 446 participants completed the study at T1. We excluded 30 participants because of missing data or failing at least one of two attention checks (instructed questionnaire answers) and 10 due to a self-reported OCD diagnosis. We excluded the latter to not bias our non-clinical spectrum approach as most recent findings showed distinct psychiatric score developments in patients versus non-patients during the pandemic^17^. This resulted in a final T1 sample of 406 participants (233 females; *M*_age_ = 34, *SD*_age_ = 12.613).

We re-invited participants to also take part at T2. Of the final T1 sample, 315 completed this second time point, of which we excluded additional 19 participants because of missing data or failed attention checks. The final T2 sample consisted of 296 participants (164 females; *M*_age_ = 35, *SD*_age_ = 12.623; cf. Supplementary Table 1 for sample characteristics). This led to an overall retention rate of 78%. We did not observe any differences in mental-health symptoms (at T1) between subjects who did versus did not participate in the T2 study as indicated by Welch’s two sample *t*-tests (OC symptoms: *t*(404) = 1.588, *p* = 0.113; anxiety: *t*(404) = 1.790, *p* = 0.074; depression: *t*(404) = 1.498, *p* = 0.135). As only one participant indicated a positive Covid-19 test at T1 and two at T2, we did not exclude these subjects or controlled for this status in the subsequent analysis as the small number of subjects is unlikely to alter our results.

### Questionnaires

We implemented all questionnaires using a web API programmed with React JS libraries (https://reactjs.org/).

#### Standardized questionnaires

To assess the impact of the Covid-19 pandemic on OC, anxiety, and depression symptoms, we administered self-report psychiatric questionnaires. We assessed OC symptoms using the Padua Inventory-Washington State University Revision (PI-WSUR)^35^. We chose the PI-WSUR because of its good test–retest reliability^38^ and detailed subscales. These subscales enabled us to look at changes in scores over repeated testing in a fine-grained manner. We further created a new subscore excluding items that might have been biased by the pandemic context (e.g. ‘If I touch something I think is ‘contaminated’, I immediately have to wash or clean myself’). Control analyses based on this score should ensure that our results were not merely driven by pandemic-related adaptive behaviours captured by PI-WSUR items evolving around hygiene and disease (cf. Supplementary Table 2 for item classifications and accompanying text for score validation).

We further measured anxiety and depression using the Hospital Anxiety and Depression Scale (HADS)^36^. We did so because these dimensions show substantial overlap in the general population^39,40^, and they are often comorbid in patients with OCD^12^. The HADS consists of two subscales (anxiety and depression) that are intended to be evaluated separately. We chose the HADS scale as it solely focuses on psychological anxiety and depression symptoms in contrast to other popular scales^41,42^, which include items related to physical symptoms (e.g. insomnia) that may be caused by physical illness (such as Covid-19). The HADS also has a high internal consistency and test–retest reliability^43^.

#### Development and validation of the Covid-19-related information-seeking questionnaire

To investigate information seeking during the pandemic and across different psychiatric domains, we developed a new Covid-19 information-seeking questionnaire. The questionnaire entailed five items with a 5-point Likert scale with lower scores indicating less information seeking. Items asked about information exchange and seeking via different social (and) media channels (cf. Supplementary Table 3 for all items). We assessed the dimensionality of our questionnaire using a principal component analysis (PCA; cf. Supplementary Fig. 1 and Supplementary Table 4), internal consistency examining the Cronbach’s Alpha coefficient, and test–retest reliability looking at Pearson’s correlation of T1 and T2 scores (cf. Supplementary Information).

#### Baseline news and social media consumption

To control for potential confounds unrelated to the pandemic, we also asked participants to retrospectively indicate their average weekly news and social media consumption before the onset of the pandemic (i.e. November 2019; Supplementary Table 5). To ensure the robustness of our main findings, we repeated regression models assessing information seeking with their baseline news and social media consumption score as a covariate.

#### Adherence to governmental guidelines

To investigate concrete behavioural links of information seeking and psychiatric scores, we asked participants to indicate to which degree they followed recommendations from authorities (Supplementary Table 6). We administered these questions after the ease of lockdown at T2 as by then there were substantial behavioural guidelines put in place by the UK government to prevent the spread of Covid-19. In the rest of the paper, we will refer to the total score as guideline adherence score.

### Statistical analyses

We pre-processed and analysed data in MATLAB 2020a (MathWorks) and used SPSS Statistics (version 26, IBM) and R, version 3.6.2 via RStudio version 1.2.5033 (http://cran.us.r-project.org) for further data analyses.

To investigate whether psychiatric scores changed differently over time, we conducted a repeated-measures ANOVA with two within-subject factors (F1: psychiatric dimension, F2: time point), the *z*-scored covariates IQ, age and gender. We applied Huynd–Feldt correction as sphericity (*ε*) was larger than 0.75. For comparability between scores, we first *z*-scored all psychiatric scores across both time points. To further compare the changes of psychiatric scores and information seeking between time points, we conducted two-sided paired *t*-tests using the stats package in R^44^, supplemented with paired permutation tests using the CarletonStats package^45^.

To evaluate the association of Covid-19-related information seeking and psychiatric scores for each time point separately, we computed robust multiple regression models using the rlm function of the MASS package^46^ in R with a bisquare method to down-weight outliers and assessed their *p* values using the rob.pvals function of the clickR package^47^. To investigate changes in the association between information seeking and psychiatric scores over time, we also conducted a random intercept mixed-effects model using the lme4 package^48^. The model entailed main effects for time point (coded as 0 (T1) and 1 (T2)) and all *z*-scored psychiatric scores as well as interactions between time point and psychiatric scores and demographic control variables. In the syntax of the *lme4* package, the model was specified as follows:

Information seeking ~ time point * (OC symptoms + anxiety + depression) + gender + age + IQ + education + essential worker status + (1|subject)

Finally, we conducted a mediation analysis to examine whether information seeking was associated with guideline adherence. For this analysis, we used the Mediation Toolbox in MATLAB (https://github.com/canlab/MediationToolbox), which adapts the standard three-variable path model^49,50^. All results refer to two-tailed bootstrapped *p* values, and the procedure was repeated 10,000 times with replacement producing a null hypothesis distribution. In our mediation model, the total OC symptoms score at T1 was taken as a predictor, guideline adherence at T2 as the dependent variable, and information seeking at T1 as the mediator (cf. Supplementary Information for control adaptations of the hypothesized mediation model). The interpretation of our mediation results was guided by the prominent, most recent typology of the field^51^. According to this, an insignificant direct effect (path c′) combined with a significant indirect effect (path ab) is interpreted as a full mediation.

All information-seeking regression models were covaried for age, gender, education, essential worker status, and IQ. We *z*-scored all included variables except for nominal (gender: 0 = female and 1 = male; essential worker status, 1 = yes, 0 = no) and ordinal (education: highest level of education ranging from 1 to 4; cf. Supplementary Table 1 for level specifications) covariates prior to the analysis to allow comparability of regression coefficients. To ensure that multicollinearity was not driving any of our results, we examined the variance inflation factor (VIF) for all multiple regression models. The VIF was below 5 for all models and thus below the standard cutoff score of 10^52^.

## Results

To investigate the development of OC, anxiety and depression symptom dimensions during the Covid-19 pandemic, we tested a (non-clinical) general public UK sample longitudinally at two time points during and after the first pandemic wave.

### Heightened psychiatric symptom scores during the Covid-19 pandemic

**Fig. 2:**
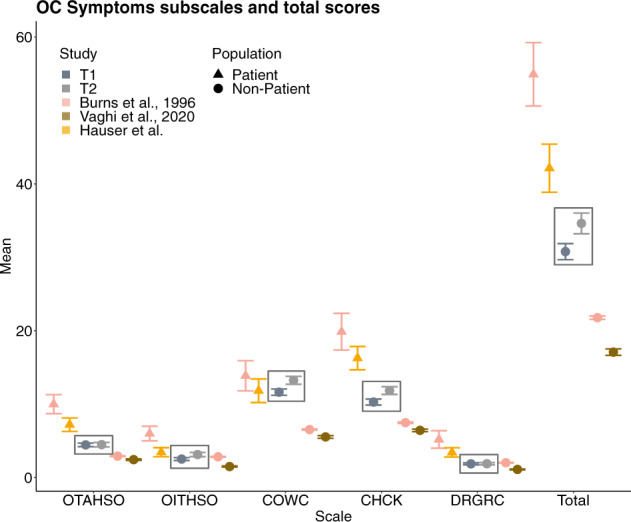
Average OC symptoms sub- and total scores of the Padua Inventory Washington State University Revision (PI-WSUR) at T1 and T2 in comparison to previous studies. Current study summary statistics are highlighted by surrounding squares (NT1 = 406; NT2 = 296). These are put in context with two pre-pandemic patient samples reported by Burns et al.^35^ (*N* = 15) displayed as pink triangles and unpublished patient data by Hauser et al. (*N* = 31), displayed as yellow triangles. A pre-pandemic non-patient sample reported by Burns et al.^35^ (*N* = 5010) is shown as pink dots and data from a previously collected young UK public sample reported by Vaghi et al.^53^ (*N* = 1606) as brown dot. The elevated scores in our sample do not imply that these participants suffer from a clinically relevant disorder, but show that symptoms are elevated across multiple subscales (including some that do not entail Covid-related items, e.g. the CHCK-score) and increased from T1 to T2. OTAHSO Obsessional Thoughts about Harm to Self or Others, OITHSO Obsessional Impulses about Harm to Self or Others, CHCK Checking Compulsions, COWC Contamination Obsessions and Washing Compulsions, DRGRC Dressing and Grooming Compulsions. Data points are means, and error bars represent standard errors.

We tested the hypothesis that the Covid-19 pandemic was associated with an overall increase in sub-clinical psychiatric symptom scores that have been linked to OCD. As expected, OC symptoms, anxiety, and depression scores were all elevated. To assess OC symptoms, we used the PI-WSUR total score, which had a mean of 30.775 (*SD* = 22.103; Fig. 2) at T1. These PI-WSUR levels are clearly higher than what has been reported in pre-pandemic population samples^35,53–55^. We restrained from quantitative comparisons due to unequal sample sizes and other potential confounding factors such as the large contextual differences (pandemic vs. non-pandemic) between the studies, which also make clinical inferences challenging. However, overall our sample seemed to score between patient and non-patient samples with some subscores being similar to pre-pandemic OCD patient samples (Fig. 2)^35^.

**Fig. 3:**
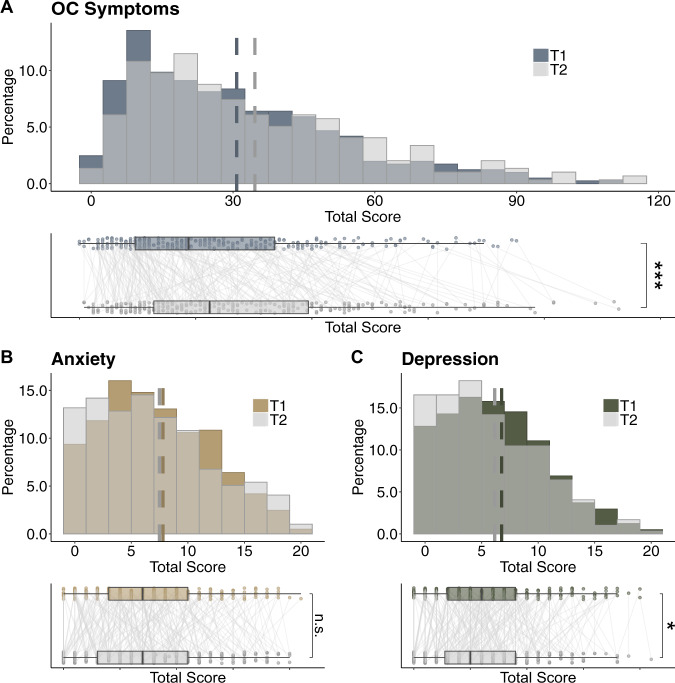
Changes of psychiatric symptoms during the first Covid-19 pandemic wave. Top-panels show the distributions of OC symptoms (**A**) as measured by PI-WSUR, anxiety (**B**) and depression scores (**C**) of the Hospital Anxiety and Depression Scale (HADS) at time point 1 (T1; *N* = 406) and time point 2 (T2; *N* = 296). Dashed lines denote the means of each time point. The bottom panels show boxplots for each of the psychiatric symptoms. Boxplots visualize an increase in OC symptom scores from T1 to T2 (**A**), stable levels of anxiety scores (**B**) and a decrease in depression scores (**C**). Connected thin lines show individual scores for T1 and T2 (*N* = 296). Paired *t*-test (two-tailed): *p < 0.05, ***p < 0.001. n.s. non-significant.

Similarly, we found high anxiety and depression scores (anxiety: *M* = 7.797, *SD* = 4.786; Fig. 3B; depression: *M* = 6.768, *SD* = 4.465; Fig. 3C). At T1, 48% (195/406) of all participants scored above the pre-pandemic cutoff score of 8 on anxiety and 41% (166/406) on depression. This means that this general UK public sample scored high on all three dimensions at the peak of the first pandemic wave, although one should be careful when directly comparing these scores with pre-pandemic comparison samples as the pandemic context may have also impacted the perception of the questionnaire items (cf. below).

#### Only OC symptoms rise further during the pandemic

We next investigated the trajectories of these psychiatric symptoms throughout the pandemic. Leveraging the longitudinal design of our study, we assessed whether symptoms showed signs of adaptation (i.e. a decrease) or whether they rose further^29,56^.

To do so, we ran a two-way repeated-measures ANOVA with within-subject factors time (T1 vs. T2) and psychiatric domain (OC, anxiety, and depression symptoms). We found a significant time by psychiatric domain interaction (*F*(1.970, 575.248) = 19.848, *p* < 0.001), extending a significant main effect of time (*F*(1, 292.00) = 4.957, *p* = 0.027).

To further inspect these effects, we compared T1 and T2 scores for each psychiatric domain separately. Importantly, we found that OC symptoms further increased during lockdown with higher average scores at T2 compared to T1 (*t*(295) = 5.294, *p* < 0.001; Fig. 3A; cf. Supplementary Information and Supplementary Fig. 2 for non-parametric tests).Next, we examined whether this held true when excluding the questionnaire items most closely linked to the pandemic to test whether our effects were driven by e.g. infection-related and hygiene behaviours (cf. Supplementary Information for information on this score). Importantly, we found the same increase in OC symptoms when only measured by pandemic-irrelevant items (*t*(295) = 2.824, *p* < 0.001), suggesting that the observed increase in OC symptoms throughout the lockdown cannot be prescribed to adaptive protective behaviours during the pandemic and was present across multiple OC domains (Fig. 2).

This increase in symptoms was unique to the OC domain. In fact, we saw a significant decrease in depression scores from T1 to T2 (*t*(295) = −0.436, *p* = 0.025; Fig. 3C). When analysing anxiety symptoms, we did not observe a significant change between T1 and T2 (*t*(295) = −0.025, *p* = 0.903; Fig. 3B). These findings thus suggest that whilst depression and anxiety symptoms adaptively decreased or remained on the same level, OC symptoms further rose throughout the lockdown.

#### Psychiatric dimension scores and information-seeking behaviour

Next, we assessed whether the psychiatric symptom scores were linked to pandemic-related behaviours. In particular, given the prior account of increased information seeking in people with high OC symptoms^30–34^, we expected individuals to engage in more Covid-related information seeking.

#### Information seeking over the course of the Covid-19 pandemic

We thus examined whether people engaged in information seeking using a newly developed and validated scale (cf. Supplementary Information). We assessed information seeking at both time points, allowing us to trace how this behaviour changed throughout the course of the pandemic. We found that information seeking decreased from T1 to T2 (*M*_T1_ = 17.043; *SD*_T1_ = 4.178; *M*_T2_ = 14.716, *SD*_T2_ = 4.320, *t*(295) = −2.108, *p* < 0.001; Supplementary Fig. 3). This means that participants engaged in Covid-related information seeking and did so primarily at the beginning of lockdown, when little was known about the pandemic.

#### OC symptoms were uniquely associated with increased pandemic-related information seeking

**Fig. 4:**
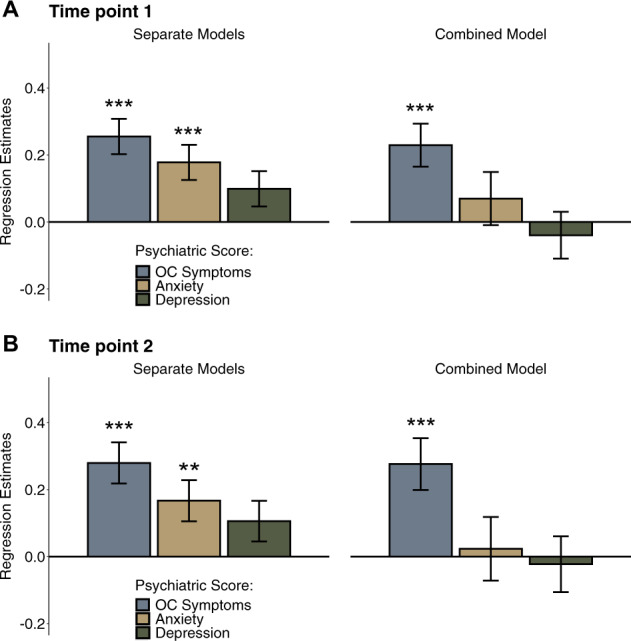
Regression analysis showing the association between Covid-19 related information seekingand psychiatric scores (OC symptoms, anxiety, and depression). (**A**, **B**) Regression models estimated separately for each psychiatric dimension (left panels) showed that OC symptoms and anxiety were associated with increased information seeking at time point T1 (*N* = 406; **A**) and time point T2 (*N* = 296; **B**). When combining all three psychiatric scores in one model (right panels) only OC symptoms remained associated with information seeking at both time points. Error bars represent standard errors and **p < 0.01, ***p < 0.001.

We next investigated whether information seeking was associated with any of the psychiatric symptom dimensions. We found that OC symptoms indeed were linked to increased information seeking at both time points (*r*_T1_(404) = 0.235, *p* < 0.001, *r*_T2_(294) = 0.249, *p* < 0.001). A similar, but smaller association was found for anxiety scores (*r*_T1_(404) = 0.182, *p* < 0.001*, r*_T2_(294) = 0.149, *p* = 0.010). We only found a weak association between depression and information seeking at T1 (*r*_T1_(404) = 0.100, *p* = 0.043), which did not hold at T2 (*r*_T2_(294) = 0.103, *p* = 0.076). All associations, except for the one with depression at T1, further remained when controlling for demographic variables (T1: OC symptoms_T1_ *β* = 0.255*, p* < 0.001; anxiety_T1_ *β* = 0.178*, p* < 0.001; depression_T1_ *β* = 0.099, *p* = 0.063; T2: OC symptoms_T2_ *β* = 0.280*, p* < 0.001; anxiety_T2_ *β* = 0.167*, p* < 0.01; depression_T2_: *β* = 0.106*, p* = 0.080; Fig. 4; cf. Supplementary Fig. 4 and accompanying text for separate models for all OC subdomains). Moreover, these results also replicated when additionally controlling for Covid-unrelated news and social media consumption (retrospective baseline measure; T1: OC symptoms_T1_ *β* = 0.239, *p* < 0.001, anxiety_T1_ *β* = 0.185, *p* < 0.001, depression_T1_ *β* = 0.117, *p* = 0.023; T2: OC symptoms_T2_ *β* = 0.243, *p* < 0.001; anxiety_T2_ *β* = 0.150, *p* = 0.012; depression_T2_ *β* = 0.118, *p* = 0.042).

These consistent correlations between psychiatric symptoms and information seeking were not surprising considering the psychiatric domains themselves are known to be related and are thus expected to show similar links (correlations between symptoms scores in this sample: OC symptoms and anxiety: *r*_T1_(404) = 0.583, *p* < 0.001, *r*_T2_(294) = 0.620, *p* < 0.001; OC symptoms and depression: *r*_T1_(404) = 0.387, *p* < 0.001, *r*_T2_(294) = 0.462, *p* < 0.001; anxiety and depression: *r*_T1_(404) = 0.684, *p* < 0.001; *r*_T2_(294) = 0.703, *p* < 0.001; Supplementary Fig. 5).

To assess which of the psychiatric domains uniquely explained increased Covid-related information seeking, we conducted a regression analysis including all psychiatric symptom scores as predictors of information seeking. This analysis revealed that only OC symptoms were uniquely associated with information seeking (*β*_T1_ = 0.229, *p* < 0.001; Fig. 4) and that there was no longer any association with anxiety (*β*_T1_ = 0.070*, p* = 0.383) or depression (*β*_T1_ = −0.040*, p* = 0.574). The same effects were found when investigating T2 scores (OC symptoms: *β*_T2_ = 0.276*, p* < 0.001; anxiety: *β*_T2_ = 0.023*, p* = 0.807). Again, including baseline news and social media consumption as covariates did not change the results (OC symptoms: *β*_T1_ = 0.199*, p* < 0.01*, β*_T2_ = 0.226*, p* < 0.01; anxiety: *β*_T1_ = 0.078*, p* = 0.316, *β*_T2_ = 0.016*, p* = 0.866). We further replicated these effects when combining the data from both time points into one mixed-effects regression model (time point: *β* = −0.535, *SE* = 0.053, *p* < 0.001; OC symptoms: *β* = 0.229, *SE* = 0.068, *p* = 0.001; cf. Supplementary Fig. 6 and accompanying text).

#### OC symptoms promote guideline adherence through increased information seeking

**Fig. 5:**
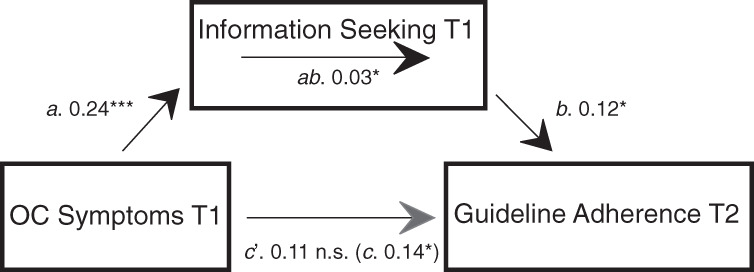
Mediation model assessing the relationship between OC symptoms and information seeking at T1 and guideline adherence at T2. The effect of OC symptoms on guideline adherence was mediated by information seeking. Displayed are standardized regression coefficients. The mediation analysis was performed on data from participants who completed the study at both time points (*N* = 296) *p < 0.05, ***p < 0.001. n.s. non-significant.

To investigate whether and how Covid-related information seeking translated into practically relevant behaviours, we also measured participants’ adherence to governmental guidelines aiming to control the pandemic once the lockdown was eased (cf. ‘Materials and methods’ and Supplementary Information for more details on this measure). We found that information seeking was indeed positively associated with guideline adherence at T2 (information seeking_T1_: *r*(294) = 0.143, *p* *= 0.014*; information seeking_T2_: *r*(294) = 0.271, *p* < 0.001). To investigate this relationship further, we performed a mediation analysis with all three factors (OC symptoms, information seeking, and guideline adherence). This analysis showed a significant direct effect of OC symptoms at T1 on guideline adherence at T2 (*β* = 0.14, *p* = 0.037, path c) and on information seeking at T1 (*β* = 0.24, *p* < 0.001, path a). It further showed a significant path from information seeking at T1 to guideline adherence at T2 (*β* = 0.12, *p* = 0.024, path b; Fig. 5). Thus, OC symptoms were associated with information seeking as well as guideline adherence, and information seeking and guideline adherence themselves were associated too. Importantly, we found a significant mediation effect via information seeking (*β* = 0.03, *p* = 0.018, path ab). When then controlling for information seeking, the effect of OC symptoms on guideline adherence was no longer significant (*β* = 0.11, *p* = 0.113, path c′). These results suggest that individuals scoring higher on OC symptoms engaged more in pandemic-related information seeking, which in turn reinforced their adherence to governmental guidelines (cf. Supplementary Information for control adaptations of this mediation model).

## Discussion

Understanding how psychiatric symptoms change throughout the Covid-19 pandemic is essential when making inferences about the crisis’ long-term mental-health consequences. We studied multiple psychiatric domains longitudinally during the first pandemic wave in the UK general public and found an elevation across all. Interestingly, while one of the psychiatric symptoms decreased and another remained stable over time, we found a further rise of OC symptoms even though the peak of the first pandemic wave had passed. Our findings highlight that OC symptoms are disproportionately affected by the pandemic by documenting their selective increase throughout the pandemic for the first time.

The documented elevations of anxiety and depression scores during the pandemic are in line with previous studies conducted in the general public^5–10^. Here, we also report a heightening in OC symptoms in the general (UK) public across multiple OC domains. Moreover, OC symptoms were inter alia elevated in domains not directly linked to Covid-related themes (e.g. increased checking vs. obsessional thoughts about harm to self or others and contamination obsessions and washing compulsions). This speaks for a generalized and thus striking effect that goes beyond adaptively increased hygiene behaviours that could be expected during the pandemic. This is most likely capturing psychological effects, e.g. elicited by the Covid-related threat or campaigns, rather than neurological consequences of a virus infection.

In contrast to previous studies that looked at the psychiatric symptoms (before and) at one time point during the pandemic^6,7^, we used a longitudinal design that allowed us to characterize the dynamics of psychiatric symptoms throughout the pandemic. Based on coping and adaptation theories^29,56^, initially developed in the field of stress research, we expected a decrease in psychiatric symptoms after the peak of the pandemic, a pattern that has recently been documented for some psychiatric domains^9^. However, we found an important dissociation in symptom dynamics. While depression decreased and anxiety levels plateaued with the ease of lockdown and the drop of Covid-19 infections, the OC symptom levels further increased. Although some environmental circumstances (e.g. lower number of infections, increased social interactions, and knowledge of the virus) might have facilitated this decrease in depression scores and kept anxiety stable, general uncertainty still remained high (e.g. due to the anticipation of a second pandemic wave, increased risk of contagion through increased social interaction) and therefore external factors do not seem to explain why one psychiatric domain would change substantially differently than the others.

We further investigated how OC symptoms related to daily behaviours such as information seeking and adherence to governmental Covid guidelines. Previously, increased information seeking has been reported in subjects with high OC symptoms in abstract laboratory tasks^30–34^. We developed a novel information-seeking measure related to Covid-19 and found that individuals with high OC scores again tended to seek more information, even when controlling for other psychiatric symptoms, the general news and media consumption and demographic variables. Furthermore, this association between OC symptoms and information seeking persisted throughout the pandemic. We further show that Covid-related information seeking was linked to later adherence to governmental guidelines. We found that information seeking mediated the influence of OC symptoms on guideline adherences meaning that individuals with elevated OC symptoms adhered more strongly to guidelines as they extensively sought Covid-related information.

It is important to note that an elevation in dimensional scores should not be mistaken for a clinical diagnosis. In contrast to self-report questionnaires, such as the ones used here, clinical assessments and interviews can differentiate between other possible diagnoses and make informed decisions about the impairment and distress, additionally taking into account the given context (e.g. a pandemic). Context variations also constrain comparisons of our data to previously established benchmarks or pre-pandemic samples. Nonetheless, our longitudinal data clearly shows a rise in OC symptoms in the general public throughout the Covid-19 pandemic. Moreover, due to their high predictive validity^35,57,58^ self-report questionnaires are also commonly used in clinical contexts and serve as valuable pre-selection instruments for mental-health services^59^. These OC symptom scores in our general public data complement increased new support requests recently reported by OCD charities^60^, symptom increases in OCD patients^4,13–16^ and observations during preceding health crises^19^. We should not make clinical statements on our dimensional data, and future studies should investigate whether OCD diagnoses are on the rise amid the Covid-19 pandemic.

Our study has some unique strengths, such as its large sample size, longitudinal testing, and real-world relevance of the measures. However, it would be interesting to further investigate these associations using additional metrics, such as smartphone geolocation patterns, browser history or in-lab experiments to overcome drawbacks of self-report measures, e.g. constraints on introspective ability^61,62^. Moreover, even though our study exploits its longitudinal design to report directional effects, given our design, causality cannot directly be inferred.

Overall, this study provides a first account of OC-symptom dynamics in the general public throughout the Covid-19 pandemic and how these symptoms translate into other behaviours such as information seeking and adherence to governmental guidelines. The increase and persistence of OC symptoms stress the importance of close monitoring of the public’s mental health and substantial support to alleviate the pandemic-related psychological burden.

## Supplementary information

Supplemental Information

## Data Availability

The code and data to reproduce the main analyses are freely available in an Open Science Framework (OSF) repository, at https://osf.io/3tue7/.
