# Supplementary material for: Obsessive–compulsive symptoms and information seeking during the Covid-19 pandemic
Source: Transl Psychiatry. 2021 May 21;11:309. doi: 10.1038/s41398-021-01410-x (PMC8138954; doi:10.1038/s41398-021-01410-x)
Supplement: Supplementary file 1 — Supplemental Information [file 41398_2021_1410_MOESM1_ESM.docx]

**Supplemental Information for**

**Obsessive-compulsive Symptoms and Information Seeking During the Covid-19 Pandemic**

Alisa M. Loosen^1 ,2^, Vasilisa Skvortsova^1, 2^ & Tobias U. Hauser^1, 2^

^1^Max Planck UCL Centre for Computational Psychiatry and Ageing Research

^2^Wellcome Centre for Human Neuroimaging, University College London

**Supplemental Table 1**

*Participants’ characteristics*

| Variable | Timepoint | |
| --- | --- | --- |
|  | T1 | T2 |
| *n* | 406 | 296 |
| PI-WSUR total (*M* ± *SD*) | 30.775 ± 22.103 | 34.618 ± 24.291 |
| HADS Anxiety total (*M* ± *SD*) | 7.797± 4.786 | 7.481 ± 5.230 |
| HADS Depression total (*M* ± *SD*) | 6.768 ± 4.465 | 6.152± 4.504 |
| Gender (f/m) | (233/173) | (164/132) |
| Age (*M* ± *SD*) | 34 ± 12.613 | 35 ± 12.623 |
| IQ (*M* ± *SD*) | 8.371 ± 3.208 | 8.725 ± 3.145 |
| Covid-19+ total (count) | 1 | 2 |
| Essential worker status (count) | 83 | 69 |
| Education (count) |  |  |
| Lower school | 4 | 3 |
| High school | 160 | 104 |
| Undergraduate degree | 177 | 136 |
| Postgraduate/Professional degree or other | 65 | 53 |

*Note.* Shown are demographic characteristics and summary statistics of the Washington State University Revision (PI-WSUR) measuring obsessive-compulsive (OC) symptoms and the Hospital Anxiety and Depression Scale (HADS) at time point 1 (T1) and time point 2 (T2). IQ was estimated using the international cognitive ability resource^1^. Covid-19+ indicates that a participant had received a positive Covid-19-test. Essential workers status corresponds to the definition provided by the UK government (https://www.gov.uk/).

**A new OC symptom subscore controlling for pandemic-relevant items.**

We created a new subscore (i.e. ‘PI-WSUR pandemic-irrelevant items’) excluding items of the PI-WSUR^2^ that we considered to potentially be related to or influenced by the Covid-19 pandemic and the related governmental policies (Supplemental Table 2). This mostly affected items related to hygiene and contamination. We validated this score and found high internal consistency Cronbach’s alpha (*α*=0.904) based on the largest dataset at T1 (of participants that had completed the entire PI-WSUR and not failed the exclusion criteria cf. Methods; *N*=412). Test-retest reliability of this new score based on the final T2 sample (N=296) was also high (*r*(294)=0.776, *p*<0.001) mirroring the good validity of the total PI-WSUR total score (*α*=0.942; *r*(294)=0.813, *p*<0.001).

**Supplemental Table 2**

*Pandemic-relevancy of items on the PI-WSUR*

| Pandemic-relevance | Item |
| --- | --- |
| Yes | 1. I feel my hands are dirty when I touch money. |
| Yes | 1. I think even slight contact with bodily secretions (perspiration, saliva urine, etc.) may contaminate my clothes or somehow harm me. |
| Yes | 1. I find it difficult to touch an object when I know it has been touched by strangers or by certain people. |
| Yes/Maybe | 1. I find it difficult to touch garbage or dirty things. |
| Yes | 1. I avoid using public toilets because I am afraid of disease and contamination. |
| Yes | 1. I avoid using public telephones because I am afraid of contagion and disease. |
| Yes | 1. I wash my hands more often and longer than necessary. |
| Yes | 1. I sometimes have to wash or clean myself simply because I think I may be dirty or “contaminated”. |
| Yes | 1. If I touch something I think is “contaminated”, I immediately have to wash or clean myself. |
| Yes/Maybe | 1. If an animal touches me, I feel dirty and immediately have to wash myself or change my clothing. |
| No | 1. I feel obliged to follow a particular order in dressing, undressing, and washing myself. |
| No | 1. Before going to sleep, I have to do certain things in a certain order. |
| No | 1. Before going to bed, I have to hang up or fold my clothes in a special way. |
| No | 1. I have to do things several times before I think they are properly done. |
| No | 1. I tend to keep on checking things more often than necessary. |
| No | 1. I check and recheck gas and water taps and light switches after turning them off. |
| No | 1. I return home to check doors, windows, drawers, etc., to make sure they are properly shut. |
| No | 1. I keep on checking forms, documents, checks, etc., in detail to make sure I have filled them in correctly. |
| No | 1. I keep on going back to see that matches, cigarettes, etc, are properly extinguished. |
| No | 1. When I handle money, I count and recount it several times. |
| No | 1. I check letters carefully many times before posting them. |
| No | 1. Sometimes I am not sure I have done things which in fact I knew I have done. |
| No | 1. When I read, I have the impression I have missed something important and must go back and reread the passage at least two or three times. |
| Yes/Maybe | 1. I imagine catastrophic consequences as a result of absent-mindedness or minor errors which I make. |
| Yes | 1. I think or worry at length about having hurt someone without knowing it. |
| Yes/Maybe | 1. When I hear about a disaster, I think it is somehow my fault. |
| Yes | 1. I sometimes worry at length for no reason that I have hurt myself or have some disease. |
| No | 1. I get upset and worried at the sight of knives, daggers, and other pointed objects. |
| No | 1. When I hear about a suicide or a crime, I am upset for a long time and find it difficult to stop thinking about it. |
| Yes | 1. I invent useless worries about germs and disease. |
| No | 1. When I look down from a bridge or a very high window, I feel an impulse to throw myself into space. |
| No | 1. When I see a train approaching, I sometimes think I could throw myself under its wheels. |
| No | 1. At certain moments, I am tempted to tear off my clothes in public. |
| No | 1. While driving, I sometimes feel an impulse to drive the car into someone or something. |
| No | 1. Seeing weapons excites me and makes me think violent thoughts. |
| No | 1. I sometimes feel the need to break or damage things for no reason. |
| No | 1. I sometimes have an impulse to steal other people’s belongings, even if they are of no use to me. |
| No | 1. I am sometimes almost irresistibly tempted to steal something from the supermarket. |
| No | 1. I sometimes have an impulse to hurt defenceless children or animals. |

**A novel Covid-19-related information-seeking questionnaire**

We developed a new questionnaire measuring Covid-19 related information seeking. The questionnaire measured news and statistics consumption via different media channels and the general information exchange with other individuals. The items are displayed below in Supplemental Table 3.

**Supplemental Table 3**

Items of the Covid-19-related information-seeking questionnaire

| Now, please think of an average day during the LAST 7 DAYS and indicate your answer based on it. |
| --- |
| 1. How often do you consume TV, radio or newspapers and journals to stay informed about COVID-19 (coronavirus)?   *5-point scale ranging from “never”, “sometimes” to “a lot”* |
| 1. How often do you engage in conversations with family, friends or colleagues about COVID-19 (coronavirus)?   *5-point scale ranging from “never”, “sometimes” to “a lot”* |
| 1. How often do you use social media (e.g. Facebook, Twitter, Instagram, YouTube, WhatsApp) to gain information about COVID-19 (coronavirus)?   *5-point scale ranging from “never”, “sometimes” to “a lot”* |
| 1. How often do you visit institutional websites (e.g. WHO, governmental websites) to gain information about COVID-19 (coronavirus)?   *5-point scale ranging from “never”, “sometimes” to “a lot”* |
| 1. Overall, how often do you inform yourself about COVID-19 (coronavirus)?   *5-point scale ranging from “never”, “sometimes” to “several times a day”* |

**Validity and psychometric properties of the new pandemic-related information-seeking questionnaire**

To assess the validity of our Covid-19 information-seeking questionnaire we ran a principal component analysis (PCA) to investigate its dimensionality. After applying our exclusion criteria (cf. Methods), we used the data of all participants at T1 that had fully completed the information-seeking questionnaire [*N*=411 (*M_age_*=34, *SD_age_*= 12.596; 237 females)]. We checked assumptions for the PCA using the Kaiser-Meyer-Olkin measure of Sampling Adequacy and Bartlett’s test of the *REdaS* package^3^ in R. The Kaiser-Meyer-Olkin measure of Sampling Adequacy (KMO=0.792) ensured that the proportion of variance among our variables, that might have been caused by an underlying factor, was large enough to be meaningful. The Bartlett’s test of sphericity [Χ2(10)=604.337, *p*<0.001] indicated that the correlation matrix was not equal to an identity matrix.

We subsequently conducted the PCA using the *prcomp* function of the *stats* package^4^ and the *hornpa* package^5^ for Horn's parallel analysis^6^ in R. The resulting scree plot of the PCA suggested a one-component solution (Supplemental Figure 1 and Supplemental Table 4). Parallel analysis, in which components are retained if the associated eigenvalue is bigger than the 95^th^ of the distribution of eigenvalues derived from a random dataset, confirmed this solution. The first component accounted for 55% of the total variance validating that all items were targeting the same underlying construct. Item loadings are additionally displayed in Supplemental Table 4 showing that all items represented one underlying construct, i.e. information seeking. Importantly, the total variance explained by the first component was higher than what could have been obtained by chance.

We obtained a Cronbach’s Alpha of *α*=0.773 for the internal consistency of our questionnaire which is considered reliable in this context^7^. Finally, we measured test-retest reliability on the final T2 sample (*N*=296) using the Pearson's Correlation Coefficient (*r*(294)=0.587, *p*<0.001) suggesting a substantial test-retest reliability of our newly developed scale^8^.


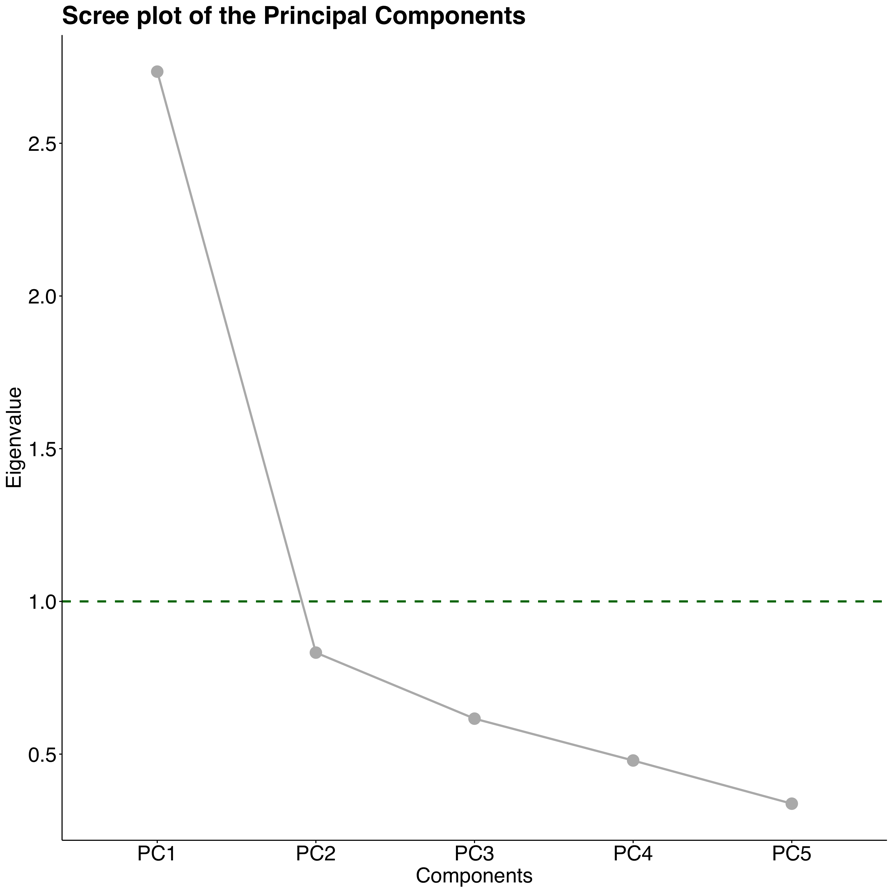


Supplemental Figure 1. **Scree plot of the Principal Components of the Covid-19 Information-seeking questionnaire.** The green dotted line marks a cut-off for Eigenvalue=1. Abbreviations: PC: Principal Component.

**Supplemental Table 4**

*Principal Component Analysis (PCA) loadings of the Covid-19 information-seeking questionnaire*

| Item | PC1 |
| --- | --- |
| Item 1 | 0.486 |
| Item 2 | 0.475 |
| Item 3 | 0.370 |
| Item 4 | 0.397 |
| Item 5 | 0.494 |

*Note.* We extracted one component (i.e. eigenvalue> 1) using PCA, which explained 55% of the total variance.

**Baseline news and social media consumption**

We controlled for baseline news and social media consumption in our analyses, to see whether our information seeking findings represented more than participants’ usual media consumption. Items are shown in the Supplemental Table 5 below.

**Supplemental Table 5**

Items measuring baseline news and social media consumption

| When reading the statements below, please think of an average day BEFORE the COVID-19 pandemic (e.g. November 2019) and indicate your answer based on it. |
| --- |
| 1. How often did you listen, read or watch the news?   *5-point scale ranging from “never”, “sometimes” to “a lot”* |
| 1. On average, how much time did you spend on social media (e.g. Facebook, Instagram, Twitter, Snapchat)? (You might want to use the screen time on your phone as an estimate if available)   Minutes: |

**Adherence to pandemic-related governmental guidelines**

We investigated the degree to which participants adhered to guidelines advocated to prevent the further spread of Covid-19. Items were collected from the National Health Service (NHS) and UK government website at the time and are shown in the Supplemental Table 6 below.

**Supplemental Table 6**

Items measuring guideline adherence

| Now, please think of an average day during the LAST 7 DAYS and indicate your answer based on it.  Please note: When we are referring to 'household' in any of the upcoming questions this also includes 'support bubbles'. Support bubbles are unions of two households that can be formed if one household consists of only one adult. |
| --- |
| 1. Do you stay at least 2 metres (6ft) away from people that are not members of your household?   *5-point scale ranging from “never”, “sometimes” to “always”* |
| 1. Do you ever meet up with more than one other household at the same time in indoor spaces?   *5-point scale ranging from “never”, “sometimes” to “very often”* |
| 1. Do you ever meet up with people from more than six different households at the same time?   *5-point scale ranging from “never”, “sometimes” to “very often”* |
| 1. Do you avoid events and locations where social distancing is not possible?   *5-point scale ranging from “not at all”, “partially” to “very much so”* |
| 1. Do you wear a face mask covering your mouth and nose when social distancing is not possible (e.g. public transport, supermarkets, restaurants)?   *5-point scale ranging from “never”, “sometimes” to “always”*   1. Do you avoid physical contact (e.g. shaking hands, hugging) with people outside of your household?   *5-point scale ranging from “not at all”, “partially” to “very much so”*   1. Do you try to limit the number of people you see outside of your household?   *5-point scale ranging from “not at all”, “partially” to “very much so”*   1. Do you avoid public transportation where possible?   *5-point scale ranging from “not at all”, “partially” to “very much so”*   1. When asked by a venue or business you visit, do you leave your real name and contact details to support the COVID-19 Test and Trace service?   *5-point scale ranging from “never”, “sometimes” to “always”*   1. Do you make sure to stay at home as much as possible?   *5-point scale ranging from “not at all”, “partially” to “very much so”*   1. Overall, do you follow the recommendations from authorities to prevent the spread of COVID-19 (coronavirus)?   *5-point scale ranging from “not at all”, “partially” to “very much so”* |

**Differential changes in psychiatric scores throughout the pandemic**

To evaluate the robustness of our evidence of differential changes in psychiatric dimensions, we additionally performed permutation tests in R to compare the observed differences in means (‾x*_t1_*-‾x*_t2_*) to a reference null distribution (Supplemental Figure 2). The observed difference in mean OC symptoms of T1 and T2 was significantly greater than the null distribution (Supplemental Figure 2A; *p*<0.001), confirming the increase in OC symptom scores between the two time points. This result held true when excluding items of high relevance to the Covid-19 pandemic (Supplemental Figure 2B**;** *p*<0.001). In contrast, the difference in mean depression was significantly smaller than the null distribution (Supplemental Figure 2D; *p*=0.024), confirming a decrease in depression scores. The observed difference in mean anxiety was not significantly different from the null distribution (Supplemental Figure 2C; *p*=0.902), thus the permutation test also did not support a change in anxiety scores from T1 to T2. These results indicate that already heightened OC symptom scores further increased from mid-way through the first pandemic wave to after it, in the absence of any changes in mean anxiety scores and a contrasting decrease in mean depression scores.


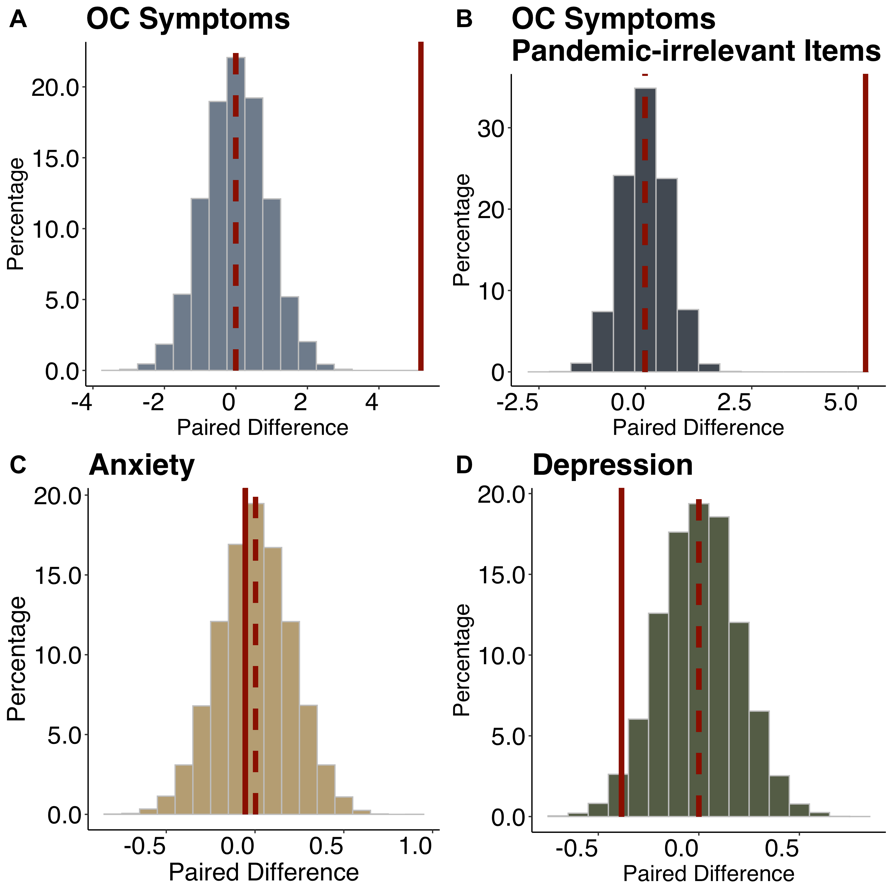


*Supplemental Figure 2.* **Histograms of the null distributions of the mean differences in psychiatric scores between T1 and T2 (*N*=20,000 permutations) for OC symptoms (A), OC symptoms as measured by pandemic-irrelevant items (B), anxiety (C) and depression symptoms (D).** The solid red lines represent the observed difference in means while the dashed red lines represents the mean difference obtained from the shuffled data. The OC symptoms pandemic-irrelevant items score was computed for each subject. OC symptom scores with and without pandemic-relevant items, as well as depression scores were more extreme than the shuffled data (Both OC scores: *p*<0.001; depression: *p*= 0.024; **A-B & D**).

**Decrease in information seeking from during the first pandemic wave to after the ease of lockdown**

Participants engaged in information seeking at both time points but less so at the second time point, after the ease of lockdown (Supplemental Figure 3A). To show the robustness of our result indicating a decrease in average information seeking from T1 to T2 we conducted an additional permutation test to compare the observed difference in means (‾x*_t1_*-‾x*_t2_*) to a reference null distribution (Supplemental Figure 3B). The observed difference in mean information seeking of T1 and T2 was significantly lower than the null distribution (*p*<0.001), confirming the decrease in information seeking from T1 to T2.


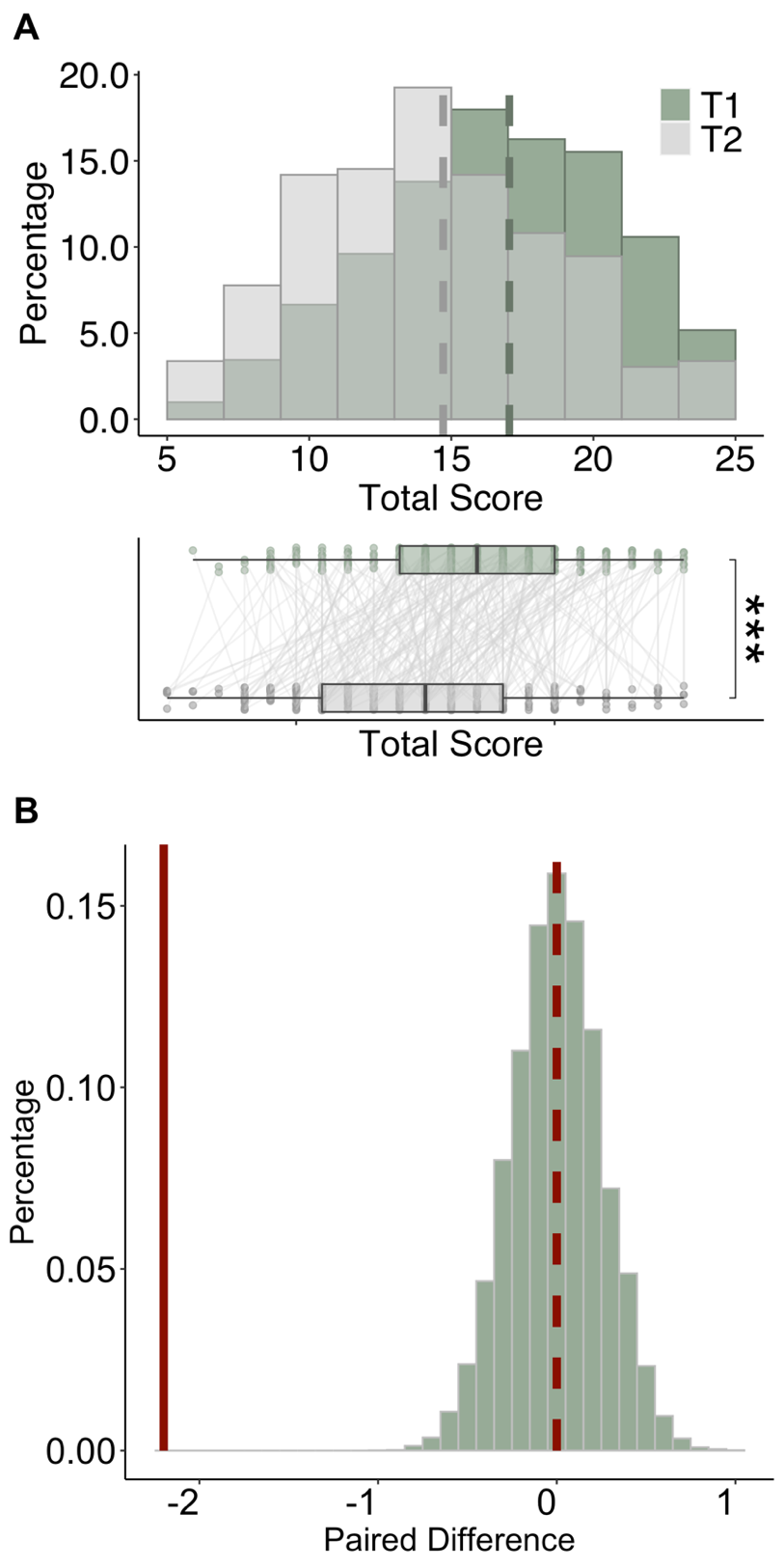


*Supplemental Figure 3.* **The spread and decrease of Covid-19 related information seeking from time point 1** (T1; *N*=406) **and time point 2** (T2; *N*=296; **A-B)**. Histogram of the Covid-19 related information seeking scores for both time points **(A).** Dashed lines denote the means of the corresponding distributions. The lower panel shows boxplots constructed with the paired data available at both time points (*N*=296). Thin lines connect individual scores between the two time points. Histograms of the null distributions of the mean differences in information seeking scores between T1 and T2 (*N*=20,000 permutations; **B**). The solid red lines represent the observed difference in means while the dashed red line represents the mean of permutation distribution. The observed ratio was significantly smaller than the permutation realizations of the ratio, which provides evidence for a decrease in information seeking from T1 to T2 (*p*<0.001). Paired t-test (two-tailed): ***- *p*< 0.001.

**Associations between PI-WSUR subscale scores and information seeking.**

To get an even more detailed look at the relationship between OC symptoms and Covid-related information seeking, we conducted separate regression models for each PI-WSUR subscore predicting information seeking at both time points, while controlling for demographic variables (cf. Methods). Using Bonferroni adjusted alpha levels of 0.010 per regression model (0.05/5), we found that the majority of subscores was linked to increased information seeking (OTAHSO: *β_T1_*=0.217, *p*<0.001; *β_T2_*=0.301, *p*<0.001; CHCK: *β_T1_*=0.214, *p*<0.001; *β_T2_*=0.271, *p*<0.001; COWC: *β_T1_*=0.279, *p*<0.001; *β_T2_*=0.211, *p*<0.01; DRGRC: *β*_T1_=0.183, *p*<0.01; Supplemental Figure 4) except for the score on “Obsessional Impulses about Harm to Self or Others”(OITHSO: *β*_T1_=-0.025, *p*=1; *β*_T2_=0.042, *p*=1) and “Dressing and Grooming Compulsions“ at T2 (DRGRC: *β*_T2_=0.124, *p*=0.248).


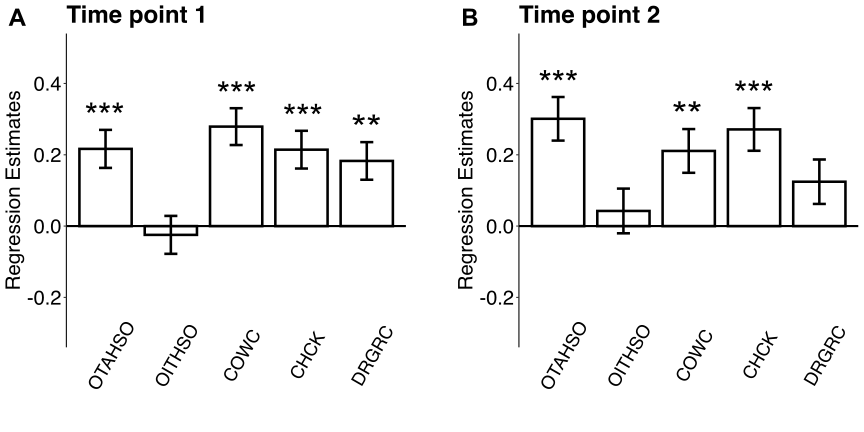


*Supplemental Figure 4.* **Regression analysis showing the association between Covid-19 related information seeking and all subscale scores of the Padua Inventory Washington State University Revision (PI-WSUR).** Regression models estimated separately for each psychiatric dimension showed that the majority of subscores, except for the OITHSO-score and the DRGRC-score at T1, was associated with increased information seeking at both time points (T1: *N*=406, **A**; T2: *N*=296, **B**). Error bars represent standard errors and* - *p*<0.05, ** - *p*<0.01, *** - *p*<0.001 corrected for multiple comparisons over the number of PI-WSUR subscales. Abbreviations: OTAHSO- Obsessional Thoughts about Harm to Self or Others, OITHSO- Obsessional Impulses about Harm to Self or Others, CHCK- Checking Compulsions, COWC- Contamination Obsessions and Washing Compulsions, DRGRC - Dressing and Grooming Compulsions.

**Psychiatric symptom scores**

Previous research has shown that OCD, anxiety and depression are highly comorbid and are usually correlated in dimensional samples^9–11^. We replicated these findings in our sample (Supplemental Figure 5).

*
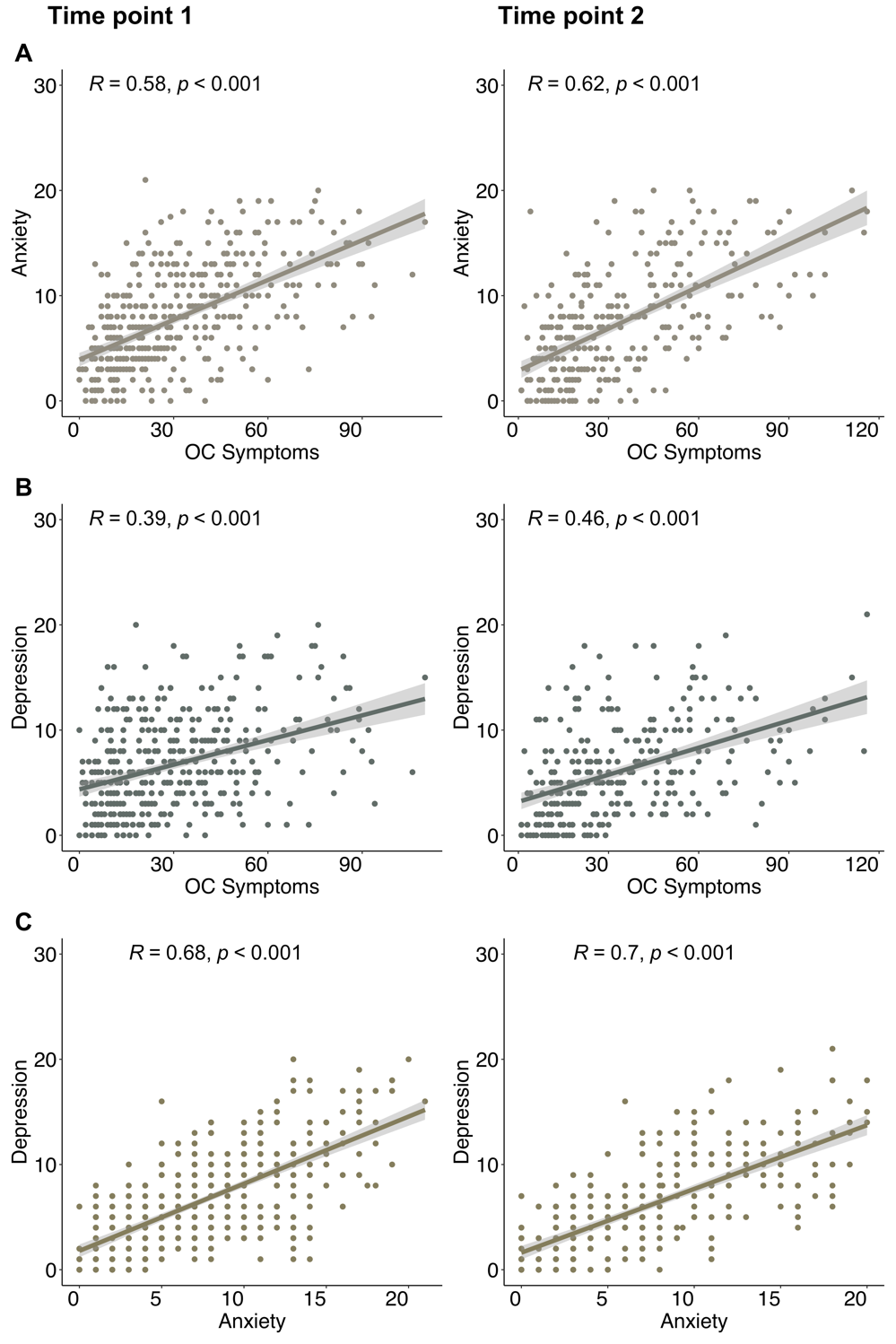
*

*Supplemental Figure 5.* **Psychiatric symptom score associations at both time points.** Pearson’s pair-wise, two-tailed correlations between psychiatric scores (OC symptoms, anxiety and depression; **A-C**) at T1 (N= 406; **left panel)** and T2 ( N= 296; **right panel)**. Shaded areas represent 95% confidence intervals.

**OC symptoms predict information seeking across both time points**

We investigated how changes in psychiatric scores related to changes in information seeking. We constructed a mixed-effects regression model with information seeking as the dependent variable and time point, psychiatric symptoms, as well as their interactions, as predictors (cf. Methods). Time point was negatively associated with information seeking (*β*=-0.536, *SE* = 0.053, *p*<0.001) confirming the observed decrease in information seeking from T1 to T2 (Supplemental Figure 6). We further found a main effect of OC symptoms showing that they were linked to higher Covid-related information seeking (*β*=0.230, *SE* = 0.068, *p*=0.001). No significant interaction effects were found between the psychiatric factors and time point (time point*OC symptoms: *β*=-0.017, *p*=0.806, time point*anxiety: *β*=-0.004, *p*=0.969, time point*depression: *β*=-0.008, *p*=0.913). These results are in support of OC symptoms as an important predictor for information seeking related to the pandemic.

*
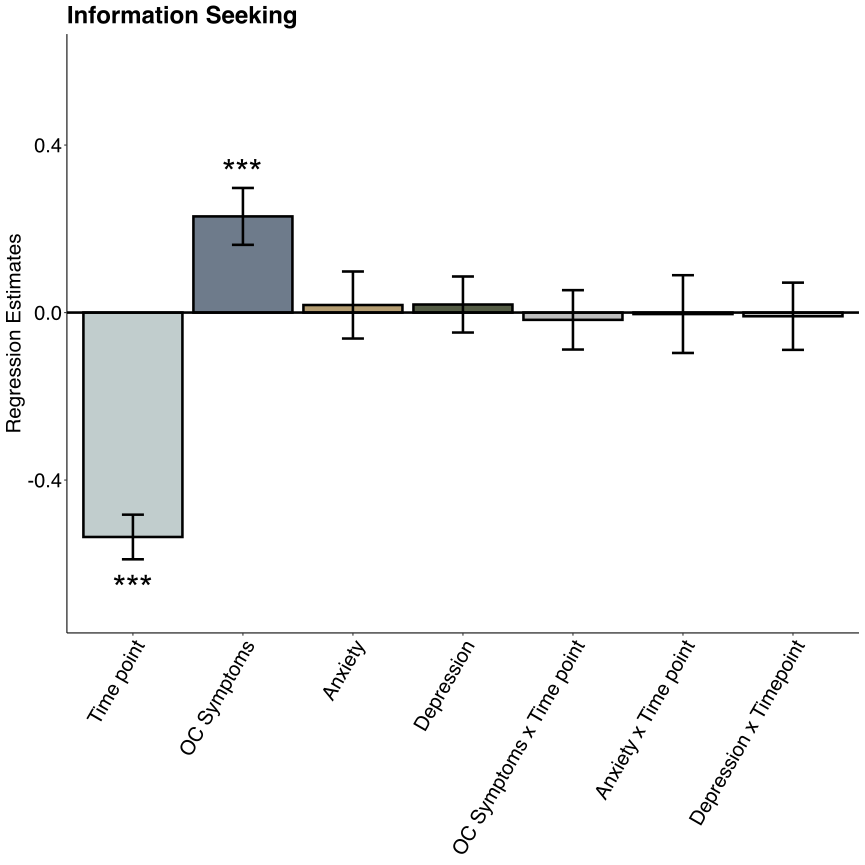
*

*Supplemental Figure 6.* **Regression analysis showing the association between Covid-19 related information seeking and psychiatric scores (OC symptoms, anxiety and depression) over time.** Estimates from a mixed-effects regression model assessing the effect of psychiatric scores, time point and their interactions on information seeking showed that time point was negatively associated with information seeking (*N*=296). This indicates that information seeking significantly decreased from T1 to T2, and OC symptoms remained positively associated with information seeking. None of the remaining main and interaction effects (OC symptoms x Time point, Anxiety x Time point, Depression x Time point) was significant. Error bars represent standard errors and *** - *p*<0.001.

**Additional mediation models**

To test the robustness of our hypothesized mediation model (investigating the association between OC symptoms, information seeking and guideline adherence) and control for correlation effects, we ran two additional mediation models. The results of our hypothesized model were replicated when using information seeking at T2 as a mediator (path *c*: *β*=0.14, *p*=0.037; path *a*: *β*=0.22, *p*<0.001; path *b*: *β*=0.24, *p*<0.001; path *ab*: *β*=0.05, *p*<0.001; path *c’*: *β*=0.08, *p*=0.209). Thus, OC symptoms as well as information seeking were associated with guideline adherence at T2 but the relationship between OC symptoms and guideline adherence was mediated by information seeking. The effect of OC symptoms on guideline adherence became non-significant when controlling for information seeking.

The model to control for directionality by reversing OC symptoms and information seeking did not show a significant mediation effect (*β*=0.02, *p*=0.088; path *ab*) whereas the significant association between information seeking and OC symptoms (*β*=0.22, *p*<0.001; path *a*) as well as information seeking and guideline adherence (*β*=0.15, *p*=0.006, path *c; β*=0.12, *p*=0.025; path *c’*) prevailed. This shows that our hypothesized model uniquely explains the relationship between OC symptoms, information seeking and guideline adherence.

A third model with the ‘PI-WSUR pandemic-irrelevant items’ score at T1 as the independent variable, information seeking at T1 as a mediator and guideline adherence at T2 as the dependent variable, also yielded a mediated relationship (path *ab*: *β*=0.02, *p*=0.012; path *c’*: *β*=0.07, *p*=0.244). Thus, even OC pandemic-irrelevant items were associated with guideline adherence and again this relationship was mediated by information seeking.

**Supplemental References**

1. Condon, D. M. & Revelle, W. The international cognitive ability resource: Development and initial validation of a public-domain measure. *Intelligence* **43**, 52–64 (2014).

2. Burns, G. L., Keortge, S. G., Formea, G. M. & Sternberger, L. G. Revision of the Padua Inventory of obsessive compulsive disorder symptoms: Distinctions between worry, obsessions, and compulsions. *Behaviour Research and Therapy* **34**, 163–173 (1996).

3. Maier, M. J. *REdaS: Companion Package to the Book ‘R: Einführung durch angewandte Statistik’*. (2015).

4. R Core Team. *R: A Language and Environment for Statistical Computing*. (R Foundation for Statistical Computing, 2019).

5. Huang, F. *hornpa: Horn’s (1965) Test to Determine the Number of Components/Factors*. (2015).

6. Bolar, K. *STAT: Interactive Document for Working with Basic Statistical Analysis*. (2019).

7. Nunnally, J. C. *Psychometric theory*. (McGraw-Hill, 1978).

8. Akoglu, H. User’s guide to correlation coefficients. *Turkish Journal of Emergency Medicine* **18**, 91–93 (2018).

9. Hauser, T. U., Moutoussis, M., Dayan, P. & Dolan, R. J. Increased decision thresholds trigger extended information gathering across the compulsivity spectrum. *Translational Psychiatry* **7**, 1296 (2017).

10. Gillan, C. M., Fineberg, N. A. & Robbins, T. W. A trans-diagnostic perspective on obsessive-compulsive disorder. *Psychol Med* **47**, 1528–1548 (2017).

11. American Psychiatric Association. *Diagnostic and Statistical Manual of Mental Disorders*. (American Psychiatric Association, 2013). doi:10.1176/appi.books.9780890425596.
